# Women’s Knowledge and Attitudes Towards Cervical Cancer Screening in Morocco

**DOI:** 10.7759/cureus.37989

**Published:** 2023-04-22

**Authors:** Karima Bendahhou, Zineb Serhier, Samir Diouny, Karima Ouadii, Amal Barkouk, Adelin Niyonsaba, Mohamed Bennani Othmani

**Affiliations:** 1 Casablanca Cancer Registry, Ibn Rochd Hospital, Casablanca, MAR; 2 Medical Informatics Laboratory, Hassan II University, Casablanca, MAR; 3 Clinical Neuroscience and Mental Health Laboratory, Hassan II University, Casablanca, MAR; 4 Clinical Neuroscience and Mental Health Laboratory, Faculty of Dentistry, Hassan II University, Casablanca, MAR; 5 Medical School, Hassan II University, Casablanca, MAR; 6 Clinical Neuroscience and Mental Health Laboratory, Faculty of Medicine and Pharmacy, Hassan II University, Casablanca, MAR

**Keywords:** cervical screening, cervical cancer screening, women’s health, womens health, morocco, attitude, knowledge, screening program, cervical cancer

## Abstract

High incidence rates of cervical cancer are still common in low- and middle-income countries (LMICs) with ineffective prevention policies. This study assessed Moroccan women's knowledge and practices regarding the cervical cancer screening program. A cross-sectional study was conducted in 2019 in four primary healthcare centers in Casablanca. Women over the age of 18 who came to these centers during the study period were invited to participate in the study. The variables collected were related to women’s knowledge of cervical cancer, the screening program, and their reasons for not participating in the screening program. The main risk factors identified by the participants were multiple sexual partners (4.3%) and sexually transmitted diseases (4%). About 77% of the cases (95% confidence interval (CI): 72.1%; 80.4%) knew that a cervical cancer screening program exists in Morocco. However, a small proportion had an idea about the population targeted by the program (46%) and the recommended interval between two screening tests (20%). Only 28% (95% CI: 19.2%; 38.2%) of eligible women had ever been screened for cervical cancer. These results underline the importance of implementing a communication strategy to increase women's awareness of the cervical screening program and their involvement in it.

## Introduction

Cervical cancer is the fourth most common cancer in women globally, with an incidence rate of 13.3 per 100,000. The total number of deaths was estimated at 341,831 in 2020, with a mortality rate of 7.3 per 100,000 [1,2].

According to the International Agency for Research on Cancer (IARC) estimates for 2020, countries in East Africa, West Africa, and Central Africa had the highest incidence rates, ranging from 31.6 per 100,000 to 40.1 per 100,000, compared to European countries and North America, where the incidence rate does not exceed 14.5 per 100,000 [2].

In Morocco, as well as in other North African countries, IARC estimated low rates compared to the rest of the African continent, with incidence and mortality rates of 6.2 and 3.7 per 100,000, respectively [2]. At the regional level, the Casablanca cancer registry reported a cervical cancer incidence rate of 10.9 per 100,000 [3] for the years 2013-2017.

Cervical cancer is a malignant tumor that occurs at the squamocolumnar junction of the cervix [4]. It takes between 15 to 20 years to develop. The main cause is the human papillomavirus (HPV) infection. In general, this infection is transitory; the virus can be eliminated in a few months without causing any lesions, but some genotypes, considered carcinogenic when they persist, lead to cervical cancer [5]. Initially, it causes precancerous lesions that may either regress to normal epithelium or evolve into carcinoma [6]. This phase, which is long enough for the development of cervical cancer, allows time to treat precancerous lesions to avoid cancer's occurrence. Thus, when preventive measures such as screening and treatment of precancerous lesions are implemented, the incidence of cervical cancer may decrease [5].

The cervical cancer burden varies markedly from one country to another, depending on the level of development and the actions taken in each country. Thus, a very significant decrease in the incidence rate has been noted over the years in developed countries. In contrast, an increase in incidence and mortality rates was reported in developing countries [7]. This heterogeneity in terms of cervical cancer incidence rates around the world indirectly reflects the efforts deployed by countries to screen and raise awareness among different populations [7]. These efforts depend on the level of development in the country. Thus, high incidence and mortality rates have been reported in countries with a low human development index (HDI). Indeed, Sigh et al. reported an association between HDI and the risk of developing or dying from cervical cancer. These risks show a downward trend of 20% and 33%, respectively, when the HDI rises by 0.2 units [8]. It should be noted that these indicators may also be different when comparing countries with low HDIs, suggesting the existence of other factors that may partially explain this difference, including HPV prevalence, the existence or not of a screening program, and its performance. In the Nordic countries [9], an important decrease has been observed in cervical cancer incidence rates over time. This is attributed in most cases to effective screening for precancerous lesions through well-organized and population-based screening programs. In contrast, in countries such as India, where no screening programs exist, the decline in the incidence rate of cervical cancer can be attributed primarily to the improved education level and awareness among women. This leads to a lower risk of exposure to risk factors such as HPV and others [10].

In Morocco, after the establishment of its first National Cancer Plan (NCP), a population-based screening program was implemented in 2010 to join the few low- and middle-income countries that had such a program [11]. It targets women between the ages of 30 and 49 using visual inspection with acetic acid (VIA) as a screening method. The invitation of women to be screened is not done systematically; eligible participants are invited accidentally when they come to the primary healthcare centers for different reasons or when their gynecologist requires it [12]. The evaluation of the cervical cancer screening program in Morocco by IARC revealed some weaknesses, including the coverage rate of the population targeted by the program. In 2016, this rate did not exceed 7.7% [13]. Several studies [14,15] have indicated that several factors could be attributed to the low coverage rate. These include socio-demographic, socio-cultural, and psychological factors, as well as the degree of awareness of cervical screening. A screening program can only be effective if the population is aware of its existence and is willing to adhere to it. Thus, the objective of the present study was to evaluate women's awareness and practices of cervical cancer screening in primary healthcare centers in Casablanca.

## Materials and methods

A cross-sectional study was conducted in 2019 in four primary healthcare centers selected randomly from two municipalities in Casablanca City (Anfa and Ain Chok). Women aged 18 and over who were present at the time of the study were invited to participate. Oral consent was requested from all participants. A questionnaire in French was used, as it is the language of instruction in scientific and medical fields in our country (Appendix 1). This questionnaire was administered to the participants by a trained medical student who is fluent in French and Moroccan dialects. The questions were instantly translated from French to the Moroccan dialect at the time of the study. It is difficult to write in the Moroccan dialect, which is easy to speak but difficult to write. It should also be noted that the dialect is very different from the Modern Standard Arabic (MSA) language, which is not understood by people with a low background in education. The questionnaire included information about age, marital status, occupation, and educational background. Additionally, women’s knowledge about cervical cancer symptoms and risk factors was collected. Participants were also asked about the existence of a family history of cervical cancer, their knowledge about the screening program and the use of the screening test, as well as the barriers to cervical cancer screening. Other questions related to the participant’s knowledge and practice of breast cancer screening were asked for the sake of comparing the two programs.

Statistical analysis was performed using Jamovi 2.2.2 software. Quantitative variables were described by their means and standard deviations. Categorical variables were described by calculating proportions with 95% confidence intervals. The percentage of women who performed the screening test was calculated for those aged 30 and older who have been married before (married, divorced, or widowed). The associations between the different variables were assessed using the Chi-square test or Fisher's exact test in cases of small expected counts.

## Results

A total of 400 women participated in the study, with an average age of 43.2 years and a standard deviation of 14.3 years. About 75% were married, and the average number of children was 2.6 (with a standard deviation of 1.9). More than 29% of the participants had a primary level of education, and 10.8% had a higher level of education. The majority of participants were housewives (87.8%). About 15% reported a family history of cervical cancer (Table 1).

**Table 1:** Socio-demographic characteristics of participants

	Number	Percentage (%)
Age mean (standard deviation)	43.2 (14.3)	
Marital status		
Married	303	75.8
Widowed	42	10.6
Divorced	28	7.0
Single	27	6.8
Number of children mean (SD)	2.6 (1.9)	
Educational level		
Illiterate	102	25.5
Primary	118	29.5
Secondary	65	16.3
High school	66	16.5
University level	43	10.8
Other	6	1.5
Occupation		
Housewife	351	87.8
Employee	29	7.2
Executive	12	3.0
Student	6	1.5
Other	2	0.6
Perceived socio-economic level		
Low	234	58.5
Medium	166	41.5
Residence area		
Urban	263	65.8
Rural	137	34.3
Health insurance	299	74.8
Causes for seeking medical care		
Diabetes	51	12.8
Vaccination	42	10.5
High blood pressure	35	8.7
Arthralgia	29	7.3
Contraception	22	5.5
Angina	22	5.5
Fever	21	5.2
Flu syndrome	19	4.8
Rash	18	4.5
Epigastralgia	17	4.2
Other	115	28.7
Not specified	9	2.3
Family history of cervical cancer	60	15.0

The participants cited metrorrhagia, leucorrhoea, and pelvic pain as the most common symptoms of cervical cancer, with 83.4%, 63.3%, and 61.5%, respectively. However, a small proportion of participants were able to cite some risk factors, such as multiple sexual partners (4.3%) and sexually transmitted infections (4.0%). The majority of the participants (91%) considered cervical cancer curable, and 76.5% (95% CI: 72.1%; 80.4%) reported being aware of the cervical cancer screening program. About 46% of participants correctly answered the age range targeted by the program, and only 20% knew the interval between two screening tests in case of negative results. In the majority of the cases, participants did not report any source of information for their knowledge about the program, and only in 12.8% and 10% of the cases, women reported that their source was health professionals and family or friends, respectively (Table 2).

**Table 2:** Participants’ knowledge of cervical cancer and the screening program (n=400)

	Number	Percentage (%)
Recognized symptoms (n=169)		
Metrorrhagia	141	83.4
Leucorrhoea	107	63.3
Pelvic pain	104	61.5
Dyspareunia	51	30.2
Risk factors cited by participants		
Multiple sexual partners	17	4.3
Sexually transmitted infection	16	4.0
Defective intimate hygiene	18	4.5
Heredity	6	1.5
Oral contraception	4	1.0
Immunodepression	2	0.5
Multiparity	1	0.3
Early sexual intercourse	1	0.3
Source of information (n=169)		
Family and friends	124	73.4
Media (radio and television)	39	23.1
Internet	24	14.2
Healthcare professional	15	8.9
Believing that cervical cancer can be cured if diagnosed early	363	90.8
Aware of the cervical cancer screening program	306	76.5; 95%CI (72.1-80.4)
The correct answer regarding screening interval	80	20.0; 95% CI (16.4-24.2)
The correct answer for the targeted age range	183	45.8; 95% CI (40.9-50.6)
Source of information on the program		
No source	271	67.8
Health professional	51	12.8
Family and friends	40	10.0
Media (radio, television)	30	7.5
Internet	8	2.0
Know someone who has been screened for cervical cancer	112	28.0
Relationship to the screened person		
Friend/neighbor	44	38.9
Sister	27	23.9
Mother	20	17.7
Daughter	7	6.2
Aunt	3	2.7
Other	12	10.6

About 28% (95% CI: 19.2%; 38.2%) of the participants, aged 30 or more, were screened for cervical cancer, and 56.5% had performed the VIA test. Of the 23 women who had been screened for cervical cancer previously, 52% were tested in a primary healthcare center in the public sector, and only 34.8% were tested in the private sector. The percentage of positive results was 8.7% (Table 3).

**Table 3:** Proportion of eligible participants who underwent screening and its circumstances VIA: visual inspection with acetic acid

	Number	Percentage (%)
Previously screened (n=83)	23	27.7 IC_95%_ [19.2-38.2]
Test performed (n=23)		
VIA	13	56.5
Pap smear	10	43.5
Date of last screening (n=23)		
> 3 years	15	65.2
≤ 3 years	8	34.8
Place of testing (n=23)		
Primary health center	12	52.2
Private sector	8	34.8
Pathology lab	2	8.7
Public hospital	1	4.3
Professional performing the test (n=23)		
General practitioner	12	52.2
Gynecologist	9	39.1
Nurse/ midwife	2	8.7
Reason for testing (n=23)		
Presence of symptoms	10	43.5
Personal decision in the absence of symptoms	9	39.1
Recommended by a physician	4	17.4
Test result (n=23)		
Positive	2	8.7
Negative	21	91.3

Among women over the age of 30 who had never been screened, 73% reported that they were willing to be screened for cervical cancer. The main reasons for not getting tested to date were the absence of symptoms (40%), thinking they were not eligible (23%), and not being aware of the program (23%) (Table 4).

**Table 4:** Attitudes toward and barriers to cervical cancer screening reported by eligible participants who have never been screened (n=60)

	Number	Percentage(%)
Reasons for non–participation in screening		
No symptoms	24	40.0
Considering not eligible	14	23.3
Lack of awareness	14	23.3
Anxiety and fear of having a positive result	7	11.7
Fear of the examination conditions	4	6.7
Lack of financial resources	4	6.7
Embarrassment and shame about the gynecological examination	3	5.0
Negligence	3	5.0
Agreeing to be tested	44	73.3
Thinking that the test is painful	47	78.3
Thinking that the test is expensive	6	10.0

Almost all participants (98% with a 95% CI: 95.8%; 98.8%) were aware of the breast cancer early detection program. The main sources of information reported were the media (80%) and health professionals (53%). More than one-third (37%) of women aged 40 or more had undergone a breast cancer screening test (95% CI: 32%; 42%) (Table 5).

**Table 5:** Knowledge and practices towards the breast cancer early detection program (n=400)

	Number	Percentage (%)
Aware of the breast cancer screening program	391	97.8; 95%CI (95.8-98.8)
Source of information		
Media (radio and television)	314	80.1
Healthcare professional	207	52.7
Family and friends	103	26.2
Internet	23	5.9
Previously tested for breast cancer (age≥40 years) (n=218)	117	53.7 IC_95%_ [46.8-59.9]
Date of last screening test		
>1 year	67	57.3
≤1 year	50	42.7

## Discussion

In this study, 400 participants were included to evaluate their knowledge and practices related to the cervical cancer screening program at the primary healthcare centers in Casablanca.

The results showed that a very small proportion of women were aware of cervical cancer risk factors. The main factors cited were multiple sexual partners (4.3%) and sexually transmitted infections (4%). About 77% of the cases, with a 95% CI: 72.1%; 80.4%, were aware of the cervical cancer screening program in Morocco. However, a small proportion knew the age range eligible for screening (46%) and the recommended interval between screening tests (20%). Knowledge of the targeted age is essential, especially in the absence of a system for inviting women to be screened. In this case, women must take the initiative to participate in the program by going to the primary healthcare center and asking to be screened. Otherwise, they may believe that they are not eligible, despite their knowledge of the program's existence, and therefore will not be screened. This lack of information could explain the low proportion observed of eligible women who were screened for cervical cancer (28%; 95% CI: 19.2%; 38.2%). Our findings are consistent with the results of the IARC evaluation of the Moroccan program, which reported weaknesses with a coverage rate of about 8% in 2016 [13]. Also, results published in 2015 evaluating the performance of the cervical cancer screening program one year after its implementation, based on indicators collected at the primary healthcare center level, showed a participation rate of 36% [16]. Another study carried out in May 2010 assessing the knowledge and attitudes of general practitioners towards screening programs showed that among the doctors who participated in the study, 25.5% were familiar with the VIA test [17].

The proportion of women aware of the cervical cancer screening program was 76.5%. This proportion was almost similar to that found in Estonia in 2011 (72.3%) and much higher than that reported by Abotchie et al. in Ghana in 2009 among sub-Saharan countries (<1%) [18,19]. However, it was lower compared to the proportion of women who were aware of the breast cancer early detection program (98%). This is mainly due to the communication policy adopted since the establishment of these two programs. Such a strategy puts emphasis on raising awareness about breast cancer, which is the most common cancer among Moroccan women. Indeed, for breast cancer, communication campaigns have been used based on media such as television and radio, with spots broadcast regularly during the day as well as programs on television and radio inviting experts to talk about the early detection program for breast cancer. Posters are also displayed in health centers, public institutions, on the tramway, and on street billboards. Cervical cancer has not been covered by similar measures. Women are invited to be screened at the time of their presentation in a health center or by a gynecologist in an opportunistic way and not in a systematic way.

The proportion of women aware of certain risk factors for cervical cancer was low compared to those reported in other populations, such as Estonian women, who reported multiple partners and sexually transmitted diseases as risk factors for cervical cancer in 70% and 75% of the cases, respectively (versus 4.3% and 4% in our study) [18]. This difference may be related to the type of question asked to gather information about this variable. Overall, data collection related to this knowledge was done through close-ended questions suggesting different factors, while in our study an open-ended questionnaire was used.

The proportion of women who were tested in our study (28%) was very low compared to that reported by Vakfari et al. in 2011, which was 57.9% in a population of women accompanying their relatives admitted to the emergency department of the General Hospital of Veria in a rural area of Northern Greece, and that reported in Iran, which was 52.1% in women aged between 30 and 59 years [20, 21]. It is important to note that women's adherence to cervical cancer screening programs varies from one population to another. It is related to socio-cultural factors and the type and characteristics of the program in place. Women's adherence can be assessed by the coverage proportion of the target population, which is an indicator for evaluating the effectiveness of the screening program. Most studies showed that this indicator is often high in developed countries compared to developing countries. Indeed, the coverage percentages in European countries reach 86.6% and 86.3% in Austria and Sweden, 82% in Denmark, 72.7% in Spain, and 64.8% in France. On the other hand, low percentages were reported in Bulgaria (46.8%), Belgium (41.3%), Italy (30.6%), and Portugal (23.9%) [22-25]. In Canada, the proportion of women participating in the screening program varied between 63.8% and 79.6% depending on the province between 2006 and 2008 [26]. This finding contrasts with the situation in Africa, where screening programs are often absent or in the early stages of implementation. In the few countries that have one, it is usually opportunistic, not organized, and non-population-based. This is generally due to the high cost of population-based screening, the absence of funds to sustain the program, and the political priorities of these countries, which do not encourage the development of such programs [27, 28].

Barriers to non-participation in cervical cancer screening, as reported by eligible women, were mainly the absence of symptoms (40%), thinking they were not eligible (23%), and a lack of awareness of the program (23%). The most common barriers reported in the literature were fear of cancer diagnosis, absence of symptoms, unavailability of a female screener, lack of awareness of the screening's benefits, administrative failures, inability to leave household chores, preoccupation with family problems, and lack of approval from the husband [29-31].

Our study has some limitations. We would have liked to use a representative sample of the general population, but for reasons of feasibility, we included women attending primary healthcare centers. However, an acceptable sample size was considered in order to generalize the results to women consulting all healthcare centers, which represent a significant subgroup of the general population.

## Conclusions

This study investigated women’s knowledge of and practices toward cervical cancer screening. Despite the high number of women who were aware of the screening program, the majority were not aware of the details, and only a small proportion of the eligible women underwent screening. These results highlight the need for implementing communication and outreach strategies to increase women's adherence and program coverage. Efforts should be made to enhance this screening program in order to make it organized with an invitation system for women.

## Appendices

Appendix 1

Questionnaire in English 

1.      Age:

2.      Marital status: □ Single             □ Married                □ Divorced           □ Widowed

3.      Number of children: …………….

4.      Occupation:…………………………………………………………

5.      Educational level :      □ Illiterate                     □ Primary                       □ Secondary           

                  □ High school               □ University level                 □Other

6.      Perceived socio-economic level:   □low                 □Medium                      □High      

7.      Health insurance:              □No   □Yes,            if Yes, type : …………………………

8.      Residence Area:                               □Urban       □Rural

9.      Reasons for seeking medical care:………………………………………………………

10.  Family history of cervical cancer:                 □Yes   □No                                            

11.  What symptoms of cervical cancer are you aware of?

            □ Metrorrhagia        □ Leucorrhoea          □ Pelvic pain       □ Dyspareunia

12.  What are the risk factors that you know about cervical cancer? .………………….………………………………………..………………………………………………

…………………………………………………………………………………...........................

13.  Specify your source of information:     

          □Health professional    □ Media (Radio, Television)  □ Family and friends     □Internet             

          □Others: …………………………..  □None  

14.  Do you think that cervical cancer can be cured if diagnosed early?

          □Yes   □No                                            

15.  Have you ever heard about the cervical cancer screening program?   □yes   □No

16.  What is the interval between two screening tests?    

            □1year    □3years     □5years          

17.  What is the age range covered by the program?   

             □20 to 40        □30 to 49     □30 to 60                             

18.  Specify your source of information:       

              □ Health professional    □ Media (Radio, Television)     □ Family and friends         □Internet              

              □Other:  ……………………..  □None  

19.  Have you been screened before for cervical cancer?        

                                                                 □Yes   □No   (if no go to question 30)                                                             

20.  If yes, which test did you perform?

              □VIA             □Papsmear

21.  How long has it been since your last test?   □ >3years                               □≤3years

22.  Where did you get your screening test?

               □ Private practice            □ Pathology lab        □Primary healthcare center                □Public hospital

                □Referral center

23.  Who performed the test?

               □Gynecologist        □General practitioner       □Midwife        □Nurse

24.  Why did you decide to get tested for cervical cancer?

               □Personal decision in absence of symptoms

               □Presence of symptoms

               □Recommended by a physician

                □Family history of cervical cancer

25.  What was the result?                         □Positive        □Negative

26.  If your test was positive, were you referred to a specialized facility for management?

               □Yes               □No

27.  Specify where:

               □Referral center

               □ Gynecologists in the private sector

               □Public hospital

               □University hospital

28.  Do you know someone who has been screened for cervical cancer: □Yes □ No

29.  What is the relationship to that person? …………………………………….

30.  If you have never been tested, please tell us why?

            □ Not eligible (never married, age<30) 

            □Lack of awareness

            □Neglect

            □Fear of the examination conditions

            □Shame/embarrassment of gynecological examination

            □Fear/anxiety of having cervical cancer

            □No symptoms

            □Consider screening a waste of time

31.  Would you be willing to be tested?             □Yes   □No

32.  Do you think the test is painful?                  □Yes   □No

33.  Do you think there is a charge for the test? □ Yes  □No

34.  Were you aware of the national breast cancer early detection program?

                                                                             □Yes   □No

35.  If yes, specify your source of information:       

              □ Health professional    □ Media (Radio, Television)      □ Family and friends         □Internet            

              □Others:  ……………………..  □None  

36.  Have you ever been tested for breast cancer?    □Yes         □No

37.  If yes, how long has it been since your last test?

               □ >1year            □≤1 year

Thank you for your participation.

Questionnaire in French 

1.       Age :

2.      Statut matrimonial : □ Célibataire             □ Mariée                 □ Divorcée                   □ Veuve

3.       Nombre d’enfants :                                                                                                                                

4.       Profession de la patiente :

5.       Profession du mari :

6.       Niveau d’études :           □ Analphabète             □ Primaire                  □Collège            

                            □ Lycée                          □Supérieur                 □autres 

7.       Niveau socio‐économique :  □Bas                      □Moyen                      □Elevé      

8.       Couverture sociale :              □Non                   □Oui ………………………………………….

9.       Origine de la participante :  □Urbaine                         □Rurale

10.   Motif de consultation :……………………………………………………

11.   Antécédents familiaux de cancer du col : □Oui           □Non

12.   Quels sont les symptômes du cancer du col que vous connaissez ?

□Métrorragies                □Leucorrhée                 □Douleur pelvienne                □Dyspareunie

13.   Quels facteurs de risque du cancer du col connaissez‐vous ? ……………………………………………. …………………………………………………………………………………………………………………………………………… 

14.   Préciser la source d’information :      □Personnel de santé                  □TV, Radio                      □Famille, connaissances               □Internet                                     □Autres……………………  

15.   Pensez-vous que qu’on peut guérir du cancer du col utérin si le diagnostic est précoce?    □Oui                                                        □Non

16.   Avez-vous une idée sur le programme national de dépistage du cancer du col ? □Oui   □Non

17.   Quel est selon vous l’intervalle entre 2 dépistages ?    □1an                   □3ans                 □5ans

18.   Quel est selon vous la tranche d’âge concernée par le programme ?

□20 à 40ans                                              □30 à 49ans                                               □30 à 60ans

19.   Précisez la source d’information :

□Personnel de santé,       □TV,radio         □Famille, connaissances                                  □Internet                                        □Autres………………………  □Aucune

20.   Avez‐vous déjà bénéficié d’un dépistage du cancer du col utérin ?  

           □Oui                                                                 □ Non (si non allez à la question 30)

21.   Si oui, quel est examen réalisé ?

□IVA                          □FCV (examen au laboratoire)

22.   Quelle est la date du dernier dépistage : □ >3ans                                 □≤3ans

23.   L’examen de dépistage était‐ il réalisé dans :

□ Cabinet privé             □Laboratoire d’anatomie pathologie              □Centre de santé                                                                                                                                  □Hôpital public             □Centre de référence

24.   Examen réalisé par qui ?

□Gynécologue                       □Médecin généraliste             □Sage femme               □Infirmière

25.   Quel était le motif de cet examen :

□Envie personnelle en absence de symptômes            □Présence de symptômes □Recommandation d’un médecin                                   □Atcd familial de cancer du col

26.   Quel était le résultat de l’examen ?                     □Positif                        □Négatif

27.   Si positif, étiez‐ vous orienté vers une structure spécialisée pour une prise en charge :

□Oui                                                        □Non

28.   Précisez :    

 □Centre de référence             □Gynécologue privé            □Hôpital public              □CHU

29.   Connaissez-vous quelqu’un qui a bénéficié du dépistage du cancer du col :

□Oui                                                  □ Non

30.   Quel est le lien de parenté ? …………………………………………….

31.   Si vous n’avez jamais bénéficié d’un test de dépistage, prière de nous préciser pourquoi ?

□ Non éligible (jamais mariée, âge<30) 

□Méconnaissance                                 □Négligence                      □La peur d’avoir mal        □La honte/gêne de l’examen de l’appareil génital                      □Manque de moyen financier                             □La peur/angoisse d’avoir le cancer du col                                   □Absence de symptômes □La peur des conditions de réalisation du dépistage                  □Perte de temps          

32.   Accepteriez-vous d’être examinée ?    □Oui                         □Non

33.   Pensez-vous que le test est douloureux : □Oui                         □Non

34.   Pensez-vous que le test est payant ? □ Oui                                □Non

35.   Connaissez‐ vous qu’il existe un programme national de dépistage du cancer du sein ?

                 □Oui                                                          □ Non

36.   Si oui, quelle est la source d’information :

□Personnel de santé            □ TV, radio          □ Famille, connaissance                       □Internet                                □Autres………………………………………….

37.   Avez‐vous bénéficié d’un examen de dépistage du cancer du sein ?    □Oui       □ Non

38.   Si oui : □ >1an                                                    □≤1 an

Merci pour votre participation
